# Ether cross-link formation in the R2-like ligand-binding oxidase

**DOI:** 10.1007/s00775-018-1583-3

**Published:** 2018-06-26

**Authors:** Julia J. Griese, Rui M. M. Branca, Vivek Srinivas, Martin Högbom

**Affiliations:** 1 Department of Biochemistry and Biophysics, Stockholm University, 106 91 Stockholm, Sweden; 2 Department of Cell and Molecular Biology, Uppsala University, 751 24 Uppsala, Sweden; 3 Cancer Proteomics Mass Spectrometry, Department of Oncology-Pathology, Science for Life Laboratory, Karolinska Institutet, Box 1031, 171 21 Solna, Sweden

**Keywords:** Di-metal carboxylate protein, Ferritin, Ribonucleotide reductase, R2-like ligand-binding oxidase, X-ray crystallography

## Abstract

R2-like ligand-binding oxidases contain a dinuclear metal cofactor which can consist either of two iron ions or one manganese and one iron ion, but the heterodinuclear Mn/Fe cofactor is the preferred assembly in the presence of Mn^II^ and Fe^II^ in vitro. We have previously shown that both types of cofactor are capable of catalyzing formation of a tyrosine–valine ether cross-link in the protein scaffold. Here we demonstrate that Mn/Fe centers catalyze cross-link formation more efficiently than Fe/Fe centers, indicating that the heterodinuclear cofactor is the biologically relevant one. We further explore the chemical potential of the Mn/Fe cofactor by introducing mutations at the cross-linking valine residue. We find that cross-link formation is possible also to the tertiary beta-carbon in an isoleucine, but not to the secondary beta-carbon or tertiary gamma-carbon in a leucine, nor to the primary beta-carbon of an alanine. These results illustrate that the reactivity of the cofactor is highly specific and directed.

**Electronic supplementary material:**

The online version of this article (10.1007/s00775-018-1583-3) contains supplementary material, which is available to authorized users.

## Introduction

Di-metal carboxylate proteins from the ferritin-like superfamily use a dinuclear metal cofactor to reduce oxygen and catalyze a variety of one- or two-electron redox reactions from the resulting high-valent state of the metal cluster [1–5]. While most groups of di-metal carboxylate proteins utilize a diiron cofactor, in recent years other groups have been shown to host a dimanganese or a mixed manganese/iron cofactor instead [6–8]. The heterodinuclear Mn/Fe cofactor is found in the R2 subunits of subclass Ic ribonucleotide reductases (denoted as R2c) as well as a related group of proteins known as R2-like ligand-binding oxidases (R2lox) [7]. Ribonucleotide reductases (RNRs) catalyze the reduction of ribonucleotides to deoxyribonucleotides via a radical-initiated mechanism. The R2 subunit of class I RNRs uses its dinuclear metal cofactor to generate this radical, which is reversibly transferred to the R1 subunit housing the active site for nucleotide reduction [4, 5, 9]. All three known types of di-metal carboxylate cofactors are found in class I RNR R2 proteins: the prototypical subclass Ia uses a diiron cofactor [9], class Ib and the recently proposed class Id utilize a dimanganese cofactor [10–15], whereas class Ic contains a heterodinuclear Mn/Fe center [16–19]. In class Ia and Ib R2 proteins, the metal cofactor carries out a one-electron oxidation of a nearby tyrosine residue, and the stable active state of the cofactor is ${\text{M}}_{2}^{\text{III}} - {\text{Y}} ^\cdot \left( {{\text{M}} = {\text{Mn}}\,{\text{or}}\,{\text{Fe}}} \right)$ [10, 11, 13, 20, 21]. In class Ic, however, the radical equivalent is instead stored in form of the Mn^IV^/Fe^III^ state of the cofactor [16, 22], while available data suggests that class Id forms a Mn^IV^/Mn^III^ cofactor [14, 15]. In contrast, the Mn/Fe cofactor of R2lox proteins can catalyze two-electron oxidations. Although the physiological function of R2lox proteins is not known, the cofactor catalyzes formation of a tyrosine–valine ether cross-link in the protein scaffold [23, 24]. Two proteins from this group have been characterized to date, homologs from *Mycobacterium tuberculosis* and *Geobacillus kaustophilus*. Both contain the ether cross-link, and both co-purify from heterologous expression hosts with a long-chain fatty acid ligand bound in a hydrophobic channel leading from the protein surface to the active site, hence the name ligand-binding oxidase [23, 25].

**Fig. 1:**
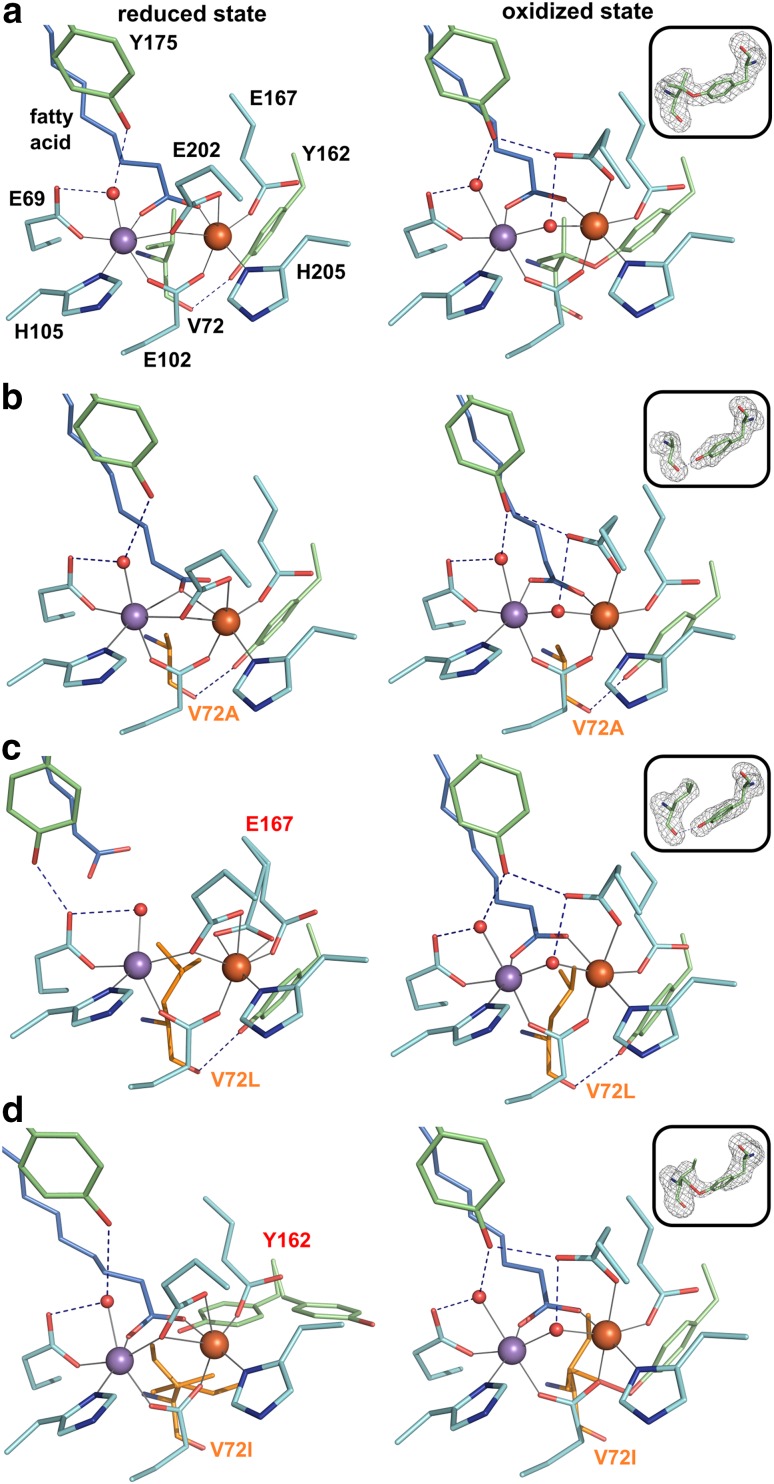
Active site structures of R2lox variants in the non-activated reduced (left panel) and oxidized resting state (right panel). All structures are shown in roughly the same orientation, with site 1 on the left. Mutated residues are highlighted in orange. Residues in alternate conformations are labeled in red. Metal–ligand bonds are indicated by grey lines, hydrogen bonds by dashed blue lines. The insets show *mF*_*o*_-*DF*_*c*_ refined omit electron density contoured at 3.0 *σ* for residues 72 and 162 in oxidized state crystals. **a** wt-R2lox (reduced state, PDB ID 4HR4; oxidized state, PDB ID 4HR0) [23]: an ether cross-link is formed between the Cβ of V72 and the phenolic oxygen of Y162 in the oxidized state. **b** V72A-R2lox: no ether cross-link is observed. **c** V72L-R2lox: no ether cross-link is formed. In the reduced state, E167 is observed in two alternate conformations, one leading to a hexacoordinate Fe ion, the other leaving one coordination site vacant (the metal–ligand bonds of both alternates are indicated). **d** V72I-R2lox: in the reduced state, both V72I and Y162 are best modeled as adopting two alternate conformations. In the oxidized state, an ether cross-link is formed between the Cβ of I72 and Y162, as indicated by the electron density and verified by mass spectrometry. The bond is unrealistically long because it is not present in all molecules in the crystal and was, therefore, not modeled in the final structure, but is indicated here for clarity. For the same reason, the isoleucine is modeled as a rotamer which best fits the density, but which it is unlikely to adopt in the cross-linked state

The Mn/Fe cofactor in R2lox self-assembles from Mn^II^ and Fe^II^ in vitro [23]. The two metal ions are bound next to each other in octahedral geometry by two histidines and four glutamates, with the manganese ion occupying the N-terminal metal-binding site 1 [23, 24] (Fig. 1a). In the reduced state, each metal ion is coordinated by one histidine and one monodentate glutamate ligand (Mn1, E69 and H105; Fe2, E167 and H205). The other two glutamates, E102 and E202, bridge the metal ions, the former in bidentate mode, the latter as bridging/chelating ligand to Fe2. The external fatty acid ligand provides another bidentate bridge, and a water molecule is bound at the open coordination site of the Mn ion in site 1. Upon oxidation, E202 shifts outwards, leaving only the monodentate coordination to Fe2, while a hydroxo ion takes its place as a bridging ligand, and an ether cross-link is formed between the Cβ of V72 and the hydroxyl oxygen of Y162 close to the active site [23, 24, 26, 27]. Cross-link formation has been proposed to be initiated from the Mn^IV^/Fe^IV^ state of the cofactor formed by oxygen reduction by abstraction of an electron from the tyrosine, with the radical subsequently transferred to the valine [23]. Alternatively, electron transfer may occur directly from the valine [28]. A second electron transfer to the metal center results in a tertiary valine carbocation, and the cross-link is formed by nucleophilic attack of the phenolic oxygen of Y162, leaving the cofactor in the observed stable resting Mn^III^/Fe^III^ state [23, 28].

We have previously demonstrated that both a Mn/Fe and a diiron cofactor, but not a dimanganese or Fe/Mn cofactor, can be formed in R2lox, and both the Mn^II^/Fe^II^ and the ${\text{Fe}}_{2}^{\text{II}}$ cofactor reduce oxygen and are chemically competent of cross-link formation [24, 29]. Here we show that Mn/Fe-R2lox forms the cross-link more efficiently than its diiron counterpart, a fact which was not observed in previous data due to the unexpected sensitivity of the cross-link to light exposure [30]. To further explore the chemical potential of the R2lox cofactor, we mutated the cross-linking valine residue (V72) and assessed the structure and cross-linking ability of the mutants. We find that the cross-link can be formed with a tertiary carbon atom in the correct position, but not with a primary or secondary carbon.

## Materials and methods

### Site-directed mutagenesis, protein production and purification

The V72A/I/L point mutations were introduced into a construct encoding full-length *Geobacillus kaustophilus* R2loxI (accession number WP_011232245) with an N-terminal hexahistidine tag in pET-46 Ek/LIC (Novagen) [23] by site-directed mutagenesis using the QuikChange Lightning kit (Agilent) and verified by DNA sequencing. All variants of R2lox were produced and purified in metal-free form as described previously [23]. Briefly, protein was produced recombinantly in *E. coli* BL21(DE3) (Novagen) grown in terrific broth (ForMedium). To obtain metal-free protein, 0.5 mM EDTA was added to the cultures immediately before induction with 0.5 mM IPTG. Apo-protein was purified via heat denaturation of contaminating proteins and nickel chelate affinity chromatography. Cells were disrupted by high-pressure homogenization in lysis buffer (25 mM HEPES-Na, pH 7.0, 300 mM NaCl, 20 mM imidazole, 0.5 mM EDTA). The lysate was cleared by centrifugation, incubated at 333 K for 10 min, and again cleared by centrifugation. The supernatant was applied to a Ni–NTA agarose (Protino, Macherey–Nagel) gravity flow column. The beads were washed with lysis buffer containing 40 mM imidazole, followed by the same buffer lacking EDTA. Protein was then eluted using lysis buffer containing 250 mM imidazole and lacking EDTA. The eluate was exchanged into storage buffer (25 mM HEPES-Na, pH 7.0, 50 mM NaCl) using a HiTrap Desalting column (GE Healthcare), concentrated to approximately 1 mM, aliquoted, flash frozen in liquid nitrogen and stored at 193 K [23]. The protein concentration was measured using experimentally determined extinction coefficients at 280 nm of 47.8 and 50.6 mM^−1^ cm^−1^ for metal-free and metal-bound protein, respectively [26].

### Crystallization and data collection

All R2lox variants were crystallized in metal-free form by vapor diffusion in hanging drops at 295 K. Wild-type and V72I-R2lox crystallized in 27.5–30% (w/v) PEG 1500, 100 mM HEPES-Na, pH 7.4–7.5. The V72A and V72L mutants nucleated neither in the wild-type condition nor upon re-screening. To obtain single crystals of these two mutants, drops were, therefore, streak-seeded with wt-R2lox crystals following a 1-h equilibration after setting up the hanging-drop plates, with the mother liquor containing 20–22.5% (w/v) PEG 1500, 100 mM HEPES-Na, pH 6.8–7.0. Crystals grew in clusters along the streak line, but could easily be separated into large three-dimensional single crystals. To reconstitute the oxidized resting state Mn/Fe cofactor in R2lox point mutants, crystals of metal-free protein were removed from their drop and soaked in mother liquor additionally containing 5 mM each MnCl_2_ and (NH_4_)_2_Fe(SO_4_)_2_ for 1–2 h under aerobic conditions and then briefly washed in 40% (w/v) PEG 1500, 100 mM HEPES-Na (at the pH of the mother liquor) before flash-cooling in liquid nitrogen. To obtain the non-activated reduced Mn/Fe cofactor, apo-protein crystals were soaked in 1 ml of 40% (w/v) PEG 1500, 100 mM HEPES-Na (at the pH of the mother liquor), 5 mM (NH_4_)_2_Fe(SO_4_)_2_, 5 mM MnCl_2_, 0.5% (w/v) sodium dithionite, 0.5 mM phenosafranin, and 0.05% (v/v) Tween 20 for 1–2 h and flash cooled directly without washing [23]. Soaking solutions were always freshly prepared immediately before use, using freshly dissolved (NH_4_)_2_Fe(SO_4_)_2_ and dithionite to ensure that the Fe was ferrous, and that oxygen was effectively removed from soaking solutions used to obtain reduced states, with phenosafranin serving as redox indicator. Data were collected at 100 K at beamline X06SA of the Swiss Light Source (SLS, Villigen, Switzerland).

### Structure determination, model building and refinement

Data were processed with XDS [31]. The structures of all R2lox point mutants were solved using the structure of the wild-type protein in the same redox state [23] not containing any ligands as a starting model. Crystals of oxidized state V72I-R2lox were obtained in the same space group as the wild-type, I222, with one molecule in the asymmetric unit, and this structure was consequently solved by Fourier synthesis. Reduced state V72I-R2lox crystals were obtained in P2_1_2_1_2, while crystals of the V72A and V72L mutants were in space group C2, all with two molecules in the asymmetric unit (Table S1). These structures were, therefore, solved by molecular replacement using Phaser in Phenix [32, 33]. Refinement was carried out with phenix.refine [32, 34] and iterated with rebuilding in Coot [35]. Refinement generally included bulk solvent corrections, individual atomic coordinate and isotropic *B* factor refinement, and occupancy refinement for alternate conformations and metal ions bound on the protein surface, but not the active site metal ions. Metal–ligand bond lengths were restrained. Solvent molecules were added with phenix.refine and manually. Hydrogens were added to the models in the later stages of refinement. One exception was made to this general protocol: in the structure of reduced state V72A-R2lox, the occupancy of the active site metal ions was refined since significant negative difference density was obtained at full occupancy of the metal ions. Structures were validated using MolProbity [36]. Data and refinement statistics are given in Tables S1 and S2. Figures were prepared with PyMOL (version 1.8.6.2, Schrodinger, LLC).

### Mass spectrometric analysis of cross-link formation

Samples were kept dark during processing. Three samples each of 100 μM apo-wt-R2lox were incubated with either four equivalents (per monomer) of MnCl_2_ and one equivalent of (NH_4_)_2_Fe(SO_4_)_2_, with the Fe salt added in steps of 0.2 equivalents every 10 min to maximize Mn/Fe cofactor formation [29], or three equivalents of (NH_4_)_2_Fe(SO_4_)_2_ only in reconstitution buffer (100 mM HEPES-Na, pH 7.0, 50 mM NaCl) under aerobic conditions for 1 h at room temperature. Similarly, two samples each of 100 µM apo-V72I/L-R2lox were incubated with two equivalents of MnCl_2_ and one equivalent of (NH_4_)_2_Fe(SO_4_)_2_. Excess metal ions were removed by passing the samples through a HiTrap Desalting column (GE Healthcare) equilibrated in storage buffer. The reconstituted protein was concentrated to 0.3–0.4 mM. From each assay replicate, 80 µg of protein were subjected to proteolytic digestion by Glu-C (Promega, enzyme:substrate ratio 1:40) in phosphate buffer (50 mM, pH 7.6) using the SP3 sample preparation method [37]. From each replicate, 300 µl of digested sample was collected and acidified by addition of 100 µl 10% formic acid (FA) prior to LC–MS. The auto sampler of a HPLC 1200 system (Agilent Technologies) injected 1 µl (approximately 200 ng of peptides) into a C18 guard desalting column (Zorbax 300SB-C18, 5 × 0.3 mm, 5 µm bead size, Agilent). Then a 15 cm long C18 picofrit column (100 µm internal diameter, 5 µm bead size, Nikkyo Technos Co., Tokyo, Japan) installed on to the nano electrospray ionization source was used. Solvent A was 97% water, 3% acetonitrile (ACN), 0.1% FA; and solvent B was 5% water, 95% ACN, 0.1% FA. At a constant flow of 0.4 µl min^−1^, a linear gradient went from 2% B up to 40% B in 45 min, followed by a steep increase to 100% B in 5 min, plateau at 100% B for 5 min, and subsequent re-equilibration with 2% B. Online LC–MS was performed using a LTQ Orbitrap Velos Pro mass spectrometer (Thermo Scientific). FTMS master scans (AGC target of 1e6) were acquired with a resolution of 30,000 and were followed by data-dependent MS/MS (AGC target of 1e5) at a resolution of 7500. In data-dependent MS/MS, the top two ions from the master scan were selected first for collision induced dissociation (at 35% energy) and afterwards for higher energy collision dissociation (at 30% energy). Precursors were isolated with a 2 *m/z* window. Dynamic exclusion was used with 60 s duration. Each sample was analyzed in technical triplicates. MS raw files were then searched against a FASTA database containing only the R2lox protein sequence using Proteome Discoverer (PD) 1.4 (Thermo Scientific). PD 1.4 can calculate protein areas based on the MS1 peak area integration of the three most abundant identified peptides. The MS1 peak area in each LCMS run of *m/z* 689.038, *z* = 3 (which corresponds to the cross-linked peptide AVIRAATVYNMIVE-AVTLD), or 693.71, *z* = 3 (which corresponds to the cross-linked peptides AVIRAATVYNMIVE-ALTLD or AVIRAATVYNMIVE-AITLD), normalized to the protein area of the same run, was used as surrogate marker for the amount of cross-link in the samples.

## Results and discussion

### A heterodinuclear Mn/Fe cofactor catalyzes cross-link formation more efficiently than a diiron cofactor in R2lox

**Fig. 2:**
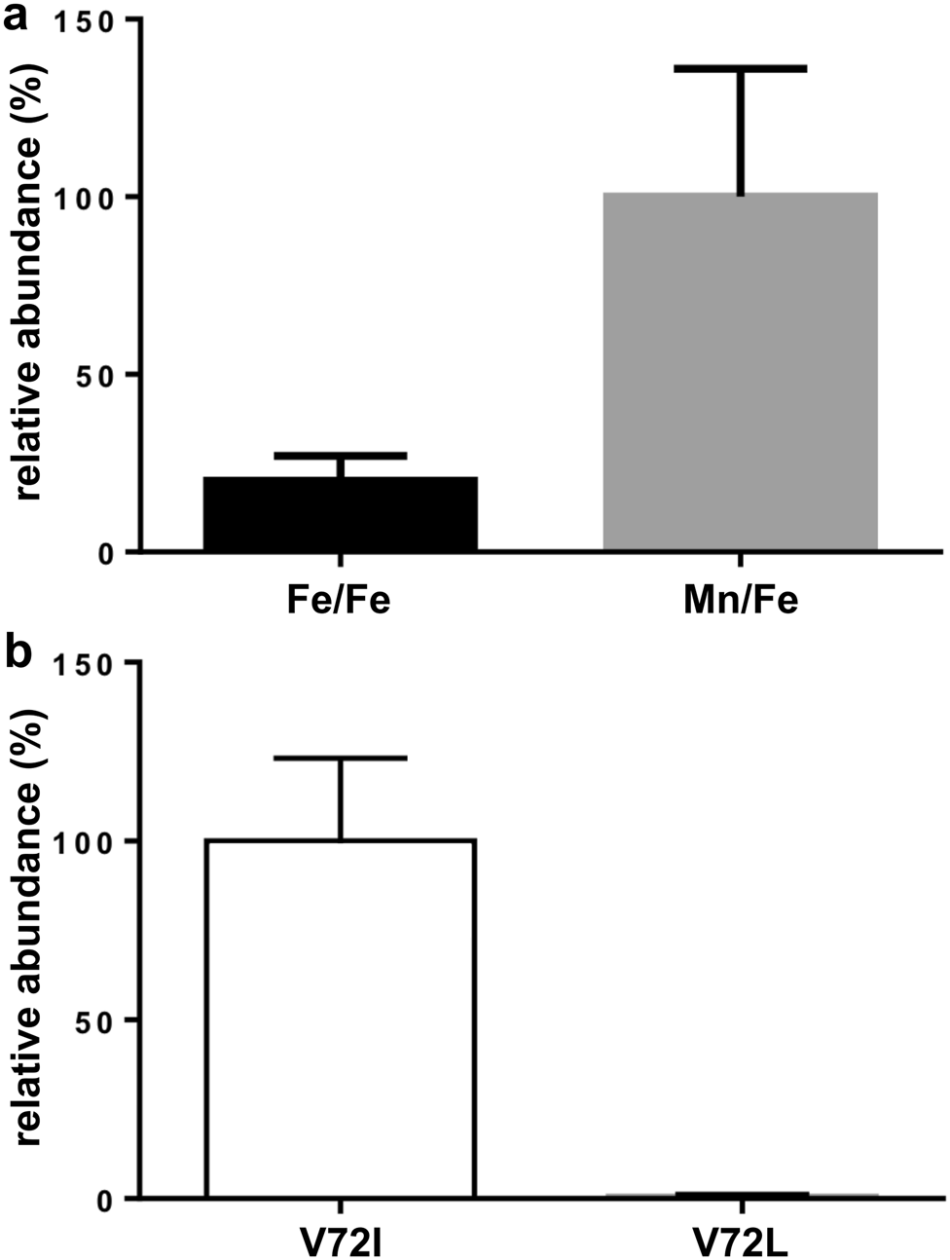
Relative abundance of the ether cross-linked peptide observed by mass spectrometry in **a** wt-R2lox aerobically reconstituted with only Fe or Mn and Fe and **b** V72I- and V72L-R2lox aerobically reconstituted with Mn and Fe. Samples were prepared in the dark. The area of the cross-linked peptide was normalized to the total protein area of the respective sample. Three (**a**) or two (**b**) replicate samples each were analyzed in technical triplicates, amounting to nine (**a**) and six (**b**) LCMS runs in total. Bars and error bars represent averages and standard deviations of the replicates

We have recently demonstrated that the tyrosine–valine ether cross-link in R2lox is broken by intense blue light, causing the metallated protein to turn purple because Y162 coordinates the metal ion in site 2 instead of E167, which is decarboxylated [30]. This observation prompted us to reexamine the reactivity of different cofactor compositions in R2lox. In a previous study we used mass spectrometry to verify that both the Mn/Fe and Fe/Fe metal centers in R2lox are capable of cross-link formation [24]. In these samples, which were prepared under light, more cross-link was observed in Mn/Fe-reconstituted R2lox than in Fe/Fe-R2lox, but the difference was not significant. Since small differences might be unobservable if some amount of cross-link was lost due to uncontrolled light exposure, we now repeated these experiments with samples kept dark during processing. The protein samples were digested with the enzyme Glu-C, which cleaves the peptide bond at the C-terminal side of glutamic and aspartic residues in phosphate buffer (50 mM, pH 7.6). The amount of cross-linked peptide detected relative to total protein was five to tenfold larger in the dark samples than in the previous samples prepared under ambient light, demonstrating that even ambient light does indeed break the cross-link to some extent [23, 24]. (Note that it is not possible to quantify the total amount of cross-link per protein due to different ionization efficiencies of the different peptides, only relative amounts of the same peptide can be compared.) Interestingly, Mn/Fe-R2lox samples contained three to fivefold more cross-linked peptide than Fe-only samples, indicating that the Mn/Fe cofactor generates the cross-link more efficiently (Fig. 2a). These data suggest that, besides incomplete formation of the ether link, breaking of the formed link due to light exposure may also be a reason for the previously observed variability of the apparent amount of cross-link in R2lox crystal structures [24].

The Mn^III^/Fe^III^ and Fe^III^/Fe^III^ cofactors in R2lox have different electronic structures, with the unique axis oriented along the Mn–water bond for the Mn^III^ ion, but along the Fe–μ–OH bond for the Fe^III^ ion in site 1, which likely leads to the Mn/Fe cofactor having a slightly more positive redox potential [26, 27]. The different electronic structures and redox potentials of the two cofactors are expected to impact their catalytic potential and lead to different reactivities. Observations on the closely related cofactor of R2c and quantum chemical calculations also suggest that the Mn/Fe cofactor is more stable in the high-valent IV/IV redox state than a diiron cluster [22, 23, 28, 38–44]. Thus, the Mn/Fe cofactor may be more efficient in initiating cross-link formation, as corroborated by the data presented here. The quantum yields for the photoconversion of Mn/Fe- and Fe/Fe-R2lox are very similar, and the site 1 metal ion is in fact not involved in the conversion process [30]. While the Mn/Fe cofactor is a more efficient catalyst for ether bond formation, the Mn/Fe-cofactored form of the protein is, therefore, not more susceptible to photoconversion than the diiron form.

To further explore the reactivity of the Mn/Fe cofactor, three different single point mutations were introduced into the R2lox scaffold. The cross-linking valine, which is completely conserved across the R2lox group [7], was changed to an alanine, isoleucine or leucine (V72A/I/L). Crystal structures were obtained of all three R2lox point mutants in the non-activated reduced and the oxidized resting state by soaking apo-protein crystals with Mn^II^ and Fe^II^ under anoxic or aerobic conditions, respectively.

### Mutation of the cross-linking valine to an alanine or leucine prevents cross-link formation

The V72A/L mutations were found to have little effect on the global structures of the reduced and oxidized resting states, but an ether cross-link was not formed in the oxidized state (Fig. 1b, c). In the wild-type, the hydroxyl group of Y162 is hydrogen-bonded to the carbonyl oxygen of V72 prior to cross-link formation [23, 24] (Fig. 1a), and this hydrogen bond is maintained in V72A/L-R2lox in the oxidized state (Fig. 1b, c). The mutations affect the position of the fatty acid ligand in comparison to the wild-type, likely due to decreased or increased steric constraints in the respective active site. Since the outward shift of E202 in the oxidized state reduces the steric strain on the active site, the effect is much more pronounced in the reduced state structures, but is also observed in the oxidized state. In the oxidized state, it appears that the bridging fatty acid ligand is slightly tilted towards residue 72 in V72A- and away from it in V72L-R2lox, although these slight differences are within the range of the coordinate error (Table S2). In reduced V72A-R2lox, however, the fatty acid ligand is less disordered than in the wild-type and clearly bridges the metal ions as a monodentate rather than a bidentate ligand, with the second carboxyl oxygen pointing towards A72 (Fig. 1b), whereas in V72L-R2lox, the fatty acid ligand is displaced upwards in the channel in the reduced state and does not coordinate the metal ions at all (Fig. 1c). Instead, E167 is observed in roughly equal proportions as the usual monodentate ligand to Fe2, leaving a vacant coordination site on this ion, and as a bidentate ligand, so that all coordination sites on Fe2 are occupied. Mn1 also has a free coordination site. Y175, which usually forms a hydrogen bond with the water molecule bound to Mn1 [24, 27], is rotated in reduced V72L-R2lox and may instead form a hydrogen bond with the unliganded carboxyl oxygen of the N-terminal metal ligand E69, the other hydrogen bonding partner of the water molecule [27]. This active site configuration mimics a conformation that is presumably also present in reduced state wt-R2lox, but in a too small proportion of molecules to be modeled with confidence, and is assumed to be the conformation that is competent for O_2_ binding and reduction, since with the fatty acid bound to the metal ions there are no free coordination sites for O_2_ [24].

### Cross-link formation is possible with an isoleucine in place of the native valine

In contrast, the bulky isoleucine side chain in V72I-R2lox has a larger effect on Y162 than on the metal-coordinating residues. It seems to have displaced the Y162 side chain, particularly in the reduced state (Fig. 1d). Both I72 and Y162 were difficult to place in the electron density. Aside from these two residues, however, the structures of V72I-R2lox are practically identical to the wild-type structures in both redox states. Interestingly, it appears as if the ether cross-link was formed between the hydroxyl group of Y162 and the Cβ of I72 in the oxidized state (Fig. 1d). Although the model with an ether linkage is more convincing than other models with different rotamers of the two residues, because the electron density is ambiguous, we used mass spectrometry to verify if the cross-link is present.

During preparation of mass spectrometry samples it was noted that the protein quickly turned purple in ambient light, whereas intense blue light is required to drive this transition efficiently in wt-R2lox. This observation is in line with previous results demonstrating that the quantum yield for photoconversion of V72I-R2lox is increased by a factor of approximately 2.5 compared to the wild-type [30]. More mass spectrometry samples were consequently prepared in the dark. The Y162-I72 cross-linked peptide was obtained by digestion of aerobically Mn/Fe-reconstituted V72I-R2lox, but only if the samples were processed in the dark, whereas the cross-link was not present in V72L-R2lox (Fig. 2b, Fig. S1 and data not shown). Therefore, an isoleucine in position 72 allows cross-link formation, but makes the link more susceptible to destruction by photoactivation. Judging from the crystal structures, it appears likely that the barrier for photoactivation is lowered due to steric hindrance caused by the bulky isoleucine sidechain, making coordination of Fe2 a more favorable position for Y162 than in the wild-type (Fig. 1d) [30].

The Mn/Fe cofactor can thus oxidize the tertiary carbon of a valine or isoleucine, but not that of a leucine, indicating that the carbon atom has to be in the correct relative position to the metal center to be oxidized. Primary or secondary carbon atoms, however, cannot be oxidized by the Mn/Fe cofactor of R2lox, likely because the resulting alkyl radical would be too unstable [23, 28, 38, 39].

## Conclusions

This study provides more insight into the chemical potential of the Mn/Fe cofactor of R2lox. We show that the heterodinuclear cofactor catalyzes cross-link formation far more efficiently than its diiron counterpart, indicating that the mixed-metal center is the biologically relevant setup. The V72 mutations demonstrate that the Mn/Fe cofactor is capable of oxidizing tertiary, but neither secondary nor primary carbon atoms. Moreover, only a tertiary beta-carbon of residue 72 is oxidized by R2lox, as the tertiary gamma-carbon of a leucine in position 72 is not. The reactivity of the heterodinuclear cofactor is thus highly specific and directed. These properties of the mixed-metal center could potentially be exploited for synthetic chemistry.

## Electronic supplementary material

Below is the link to the electronic supplementary material.

Supplementary material 1 (PDF 314 kb)

## Abbreviations

ACN: Acetonitrile
FA: Formic acid
RNR: Ribonucleotide reductase
R2c: Class Ic RNR R2 protein
R2lox: R2-like ligand-binding oxidase
wt: Wild-type
