# Supplementary material for: Ether cross-link formation in the R2-like ligand-binding oxidase
Source: J Biol Inorg Chem. 2018 Jun 26;23(6):879–86. doi: 10.1007/s00775-018-1583-3 (PMC6060897; doi:10.1007/s00775-018-1583-3)
Supplement: Supplementary file 1 — Supplementary material 1 (PDF 314 kb) [file 775_2018_1583_MOESM1_ESM.pdf]

Electronic Supplementary Material for:

Ether cross-link formation in the R2-like ligand-binding oxidase

Julia J. Griese[1,2](✉), Rui M. M. Branca[3], Vivek Srinivas[1], and Martin Högbom[1](✉)

[1]Department of Biochemistry and Biophysics, Stockholm University, SE-106 91 Stockholm, Sweden; [2]Department of Cell and Molecular Biology, Uppsala University, SE-751 24 Uppsala, Sweden; [3]Cancer Proteomics Mass Spectrometry, Department of Oncology-Pathology, Science for Life Laboratory, Karolinska Institutet, Box 1031, SE-171 21 Solna, Sweden.

✉ E-mail: julia.griese@icm.uu.se; hogbom@dbb.su.se

The Electronic Supplementary Material contains:

Table S1 Crystallographic data statistics.

Table S2 Refinement statistics.

Fig. S1 Annotated MS2 spectrum of the tyrosine-isoleucine ether cross-linked peptide in V72I-R2lox.

References

Table S1 Crystallographic data statistics

**R2lox variant**	**V72A**		**V72I**		**V72L**	
**Soaking condition**	**anoxic Mn+Fe**	**aerobic Mn+Fe**	**anoxic Mn+Fe**	**aerobic Mn+Fe**	**anoxic Mn+Fe**	**aerobic Mn+Fe**
Beamline	X06SA/SLS	X06SA/SLS	X06SA/SLS	X06SA/SLS	X06SA/SLS	X06SA/SLS
Detector	Pilatus 6MF	Pilatus 6MF	Eiger 16M	Eiger 16M	Pilatus 6MF	Pilatus 6MF
Wavelength (Å)	0.98	0.98	1.00	1.00	0.98	0.98
Resolution range (Å)	50.00-1.61 (1.71-1.61)	50.00-1.77 (1.87-1.77)	50.00-1.99 (2.11-1.99)	50.00-1.79 (1.90-1.79)	50.00-1.98 (2.10-1.98)	50.00-1.76 (1.87-1.76)
Space group	C2	C2	$P2_12_12$	I222	C2	C2
Unit cell dimensions a, b, c (Å)	161.52, 55.65, 70.05	162.46, 55.82, 69.74	127.99, 98.47, 55.86	55.58, 95.90, 127.88	160.61, 55.66, 70.08	160.61, 55.72, 70.05
β (°)	114.11	114.09	90.00	90.00	114.16	114.03
Unique reflections	67293 (8822)	53633 (7702)	48652 (7415)	32199 (4924)	37786 (5524)	52983 (6957)
Multiplicity	3.4	3.3	6.3	6.6	3.3	3.3
Completeness (%)	91.9 (75.0)	95.2 (85.1)	99.0 (95.0)	98.3 (94.7)	95.5 (88.0)	93.5 (76.5)
I/σ(I)	17.82 (1.41)	13.70 (1.20)	9.67 (1.01)	13.29 (0.78)	6.37 (1.13)	11.00 (1.10)
R_{merge} (%)	3.3 (72.4)	4.5 (78.2)	13.7 (182.8)	7.2 (212.1)	13.4 (114.2)	5.4 (79.8)
R_{meas} (%)	3.9 (86.2)	5.4 (93.6)	14.9 (199.9)	7.9 (230.8)	16.0 (136.5)	6.5 (96.0)
$CC_{1/2}$ [a]	99.9 (78.9)	99.9 (72.5)	99.7 (32.9)	99.9 (24.8)	99.3 (44.3)	99.8 (67.4)

Values in parentheses are for the highest resolution shell. Friedel pairs were merged. [a]Percentage of correlation between intensities from random half-datasets [1]. The correlation is significant at the 0.1% level in all resolution shells in all datasets.

Table S2 Refinement statistics

**R2lox variant**	**V72A**		**V72I**		**V72L**	
**Soaking condition**	**anoxic Mn+Fe**	**aerobic Mn+Fe**	**anoxic Mn+Fe**	**aerobic Mn+Fe**	**anoxic Mn+Fe**	**aerobic Mn+Fe**
PDB ID	6F6E	6F6C	6F6G	6F6F	6F6K	6F6H
Resolution range (Å)	43.57-1.63	43.57-1.77	48.59-1.99	48.09-1.79	43.57-1.98	43.53-1.76
Reflections used	66913	53956	48623	32148	37747	52956
R_{work}/R_{free} (%)[a]	17.5/20.1	18.0/22.3	19.3/24.0	17.5/20.6	19.8/26.3	18.3/23.5
Coordinate error (Å)	0.31	0.24	0.33	0.25	0.37	0.28
Non-H atoms	4887	4797	4843	2418	4724	4806
Protein residues[b]	556 (3-286/3-250;263-286)	556 (3-286/3-250;263-286)	558 (3-251;263-286/2-286)	283 (4-286)	545 (3-250;263-286/3-251;263-286)	557 (2-286/3-250;263-286)
Water molecules	236	152	136	56	161	148
Ligand molecules	2	2	2	1	2	2
Metal ions	6	6	6	3	6	6
rmsd bonds (Å)[c]	0.015	0.016	0.019	0.015	0.016	0.015
rmsd angles (°)[c]	1.075	1.041	1.209	1.116	1.206	1.038
Ramachandran favored/allowed/ outliers (%)[d]	97.3/2.5/0.2	97.3/2.7/0.0	96.7/3.3/0.0	97.5/2.5/0.0	96.8/3.0/0.2	96.9/3.1/0.0
Clashscore[d]	3.81	3.27	8.82	3.42	4.10	3.36
Wilson *B* factor ($Å^2$)	28.1	33.9	39.2	41.6	34.7	34.3
Average *B* factors ($Å^2$)[e,f]						
all atoms	38.5	44.2	53.6	54.0	43.5	47.2
protein main and side chains	39.9/36.9	44.0/44.4	52.6/54.6	54.0	42.6/44.4	46.8/47.7
site 1 metal ion	21.4/18.5	29.4/31.6	30.5/31.8	37.9	27.1/28.6	34.0/35.3
site 2 metal ion	22.6/21.4	33.7/35.2	30.5/32.2	38.6	25.7/30.7	32.7/34.5
additional metal ions	55.5/54.1	61.8/64.2	69.8/75.7	95.5	65.1/61.9	68.1/75.5
ligand	43.0/43.2	46.0/48.7	62.6/57.6	58.9	48.9/53.0	52.2/54.8
water	38.9	41.6	45.2	47.3	39.7	42.6
Occupancies <1.0 [f]						
site 1 metal ion	0.97/0.90	-	-	-	-	-
site 2 metal ion	0.88/0.91	-	-	-	-	-
additional metal ions	0.82/0.77	0.63/0.72	-	0.88	1.00/0.91	0.67/0.79

[a]R_{free} is calculated from a randomly selected subset of approximately 2000 reflections (corresponding to up to 5% of reflections) excluded from refinement. [b]Residues out of the 302 residue full-length protein included in the final model are given in parentheses. [c]Root-mean-square deviation from ideal geometry. [d]Geometry statistics were calculated with MolProbity [2]. [e]Average *B* factors were calculated with Baverage in the CCP4 suite [3]. [f]Where there are 2 molecules in the asymmetric unit, the values for chain A and chain B are given separately where applicable (A/B).

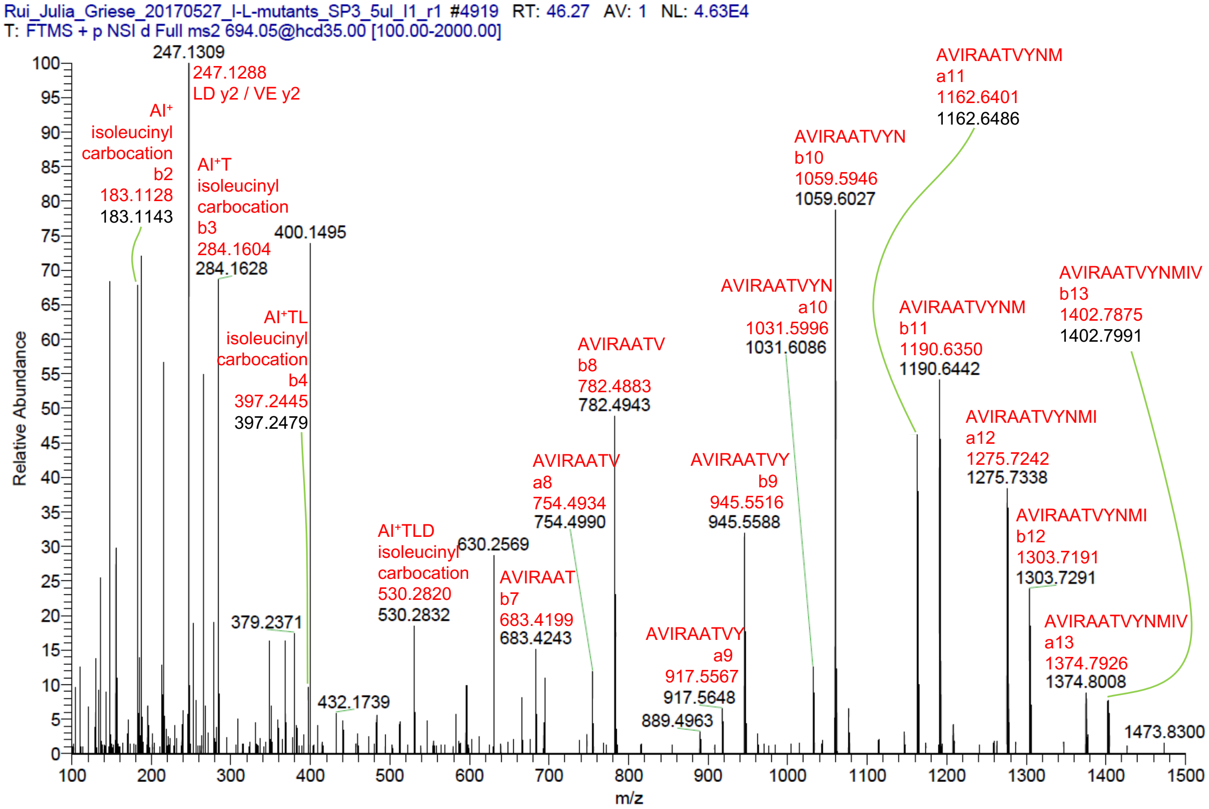

Fig. S1 Annotated MS2 spectrum of the triply charged precursor ion 693.71 m/z as found in aerobically Mn/Fe reconstituted V72I-R2lox samples. This precursor ion corresponds to the tyrosine-isoleucine ether cross-linked peptide AVIRAATV<u>Y</u>NMIVR-A<u>I</u>TLD, obtained by proteolytic digestion of V72I-R2lox with the Glu-C enzyme. Experimental m/z values are shown in black, whereas annotations and theoretical m/z values are shown in red. The mass error between observed and theoretical values is typically less than 0.01 m/z, in accordance with the resolution used (7500 at 400 m/z). Series of b and y ions are observable for both peptide chains and, more importantly, four ions containing the isoleucinyl carbocation are observed. These carbocations arise from the gas-phase cleavage (by collision induced dissociation, CID, or higher energy collision dissociation, HCD) of the ether bond between the tyrosine and isoleucine side chains

References

1. Karplus PA, Diederichs K (2012) Linking Crystallographic Model and Data Quality. Science 336:1030–1033. doi: 10.1126/science.1218231
2. Chen VB, Arendall 3rd WB, Headd JJ, et al (2010) MolProbity: all-atom structure validation for macromolecular crystallography. Acta Crystallogr Sect D Biol Crystallogr 66:12–21. doi: 10.1107/S0907444909042073
3. Winn MD, Ballard CC, Cowtan KD, et al (2011) Overview of the CCP4 suite and current developments. Acta Crystallogr Sect D Biol Crystallogr 67:235–242. doi: 10.1107/S0907444910045749
